# Pipe Resistance Loss Calculation in Industry 4.0: An Innovative Framework Based on TransKAN and Generative AI

**DOI:** 10.3390/s25123803

**Published:** 2025-06-18

**Authors:** Qinyu Zhang, Huiying Liu, Zhike Liu, Yongkang Liu, Yuhan Gong, Chonghao Wang

**Affiliations:** 1 College of Science, North China University of Science and Technology, Tangshan 063210, China; zhangqinyu@stu.ncst.edu.cn (Q.Z.); liuhuiying@stu.ncst.edu.cn (H.L.); 2 College of Electrical Engineering, North China University of Science and Technology, Tangshan 063210, China; liuzhike@stu.ncst.edu.cn (Z.L.); liuyongkang@stu.ncst.edu.cn (Y.L.); gongyuhan@ncst.edu.cn (Y.G.); 3 College of Mining Engineering, North China University of Science and Technology, Tangshan 063210, China

**Keywords:** pipeline resistance loss, attention fusion, generative artificial intelligence, KAN network

## Abstract

As the demand for deep mineral resource extraction intensifies, optimizing pipeline transportation systems in backfill mining has become increasingly critical. Thus, reducing energy loss while ensuring the filling effect becomes crucial for improving process efficiency. Owing to variations among mines, accurately calculating pipeline resistance loss remains challenging, resulting in significant inaccuracies. The rapid development of Industry 4.0 provides intelligent and data-driven optimization ideas for this challenge. This study introduces a novel pipeline resistance loss prediction framework integrating generative artificial intelligence with a TransKAN model. This study employs generative artificial intelligence to produce physically constrained augmented data, integrates the KAN network’s B-spline basis functions for nonlinear feature extraction, and incorporates the Transformer architecture to capture spatio-temporal correlations in pipeline pressure sequences, enabling precise resistance loss calculation. The experimental data collected from pipeline pressure sensors provides empirical validation for the model. Compared with traditional mathematical formulas, BP neural networks, SVMs, and random forests, the proposed model demonstrates superior performance, achieving an R^2^ value of 0.9644, an RMSE of 0.7126, and an MAE of 0.4703.

## 1. Introduction

Global mineral resource extraction has surged in response to rapid economic growth and escalating resource demands [1,2,3]. While mineral resource extraction drives economic development, it simultaneously produces massive quantities of tailings, with global annual output now surpassing 14 billion metric tons [4]. This has created an urgent imperative to develop effective tailings management strategies, a critical challenge for achieving sustainable mining operations. The filling mining method [5,6], an environmentally friendly approach, involves injecting tailing slurry into underground mining airspaces, thereby mitigating surface subsidence, enhancing mineral resource recovery, and demonstrating substantial advantages in terms of sustainable development and economic benefits. The concept of green mine construction has gained significant traction in recent years [7,8,9], further promoting the adoption of the filling mining method. Meanwhile, rapid Industry 4.0 advancements are driving digital transformation and intelligent development in mining, creating unprecedented opportunities to optimize backfill mining operations and management.

Pipeline transportation of tailing slurry constitutes a pivotal element in the overall mining process [10,11,12]. The high solids concentration and complex rheological behavior of the slurry induce significant hydraulic losses during pipeline transport, substantially reducing system efficiency. These losses directly increase energy demands and operational expenses. Through comprehensive analysis, the study determined that key influencing factors include slurry concentration, pipe diameter, and particle size distribution. In their experimental investigation, Shao et al. [13] examined the rheological properties of wind-formed sand–fly ash-based filling slurry with varying fly ash contents. Their analysis encompassed the strength development of the filling body at different ages and fly ash contents, employing both macroscopic and microscopic perspectives. The study revealed that an increase in fly ash content led to substantial changes in rheological properties, including thixotropy, plastic viscosity, and yield stress. In a related study, Zhang et al. [14] investigated the impact of mass concentration and particle size distribution on rheological parameters and slump, as well as the correlation between tailings rheological parameters, slump, and the size distribution of tailings particles. This analysis was conducted by examining the mixture of overflow and graded tailings. Yang et al. [15] employed CFD, grounded in multiphase flow theory, to investigate the effects of inlet slurry velocity, stone volume concentration, and pipe inclination on pressure drop and conveying capacity. Wang et al. [16] designed a pipe wear ring test system to determine the effect of different filling slurry ash–sand ratios and flow rates on pipe wear. They also established mathematical models between slurry density and wear rate and flow rate and wear rate. These models revealed the relationship between various influencing factors. In a related study, Qiu et al. [17] employed CFD to perform a CPB in an L-pipe, integrating the inlet velocity, viscosity, and particle size 3D network simulation of slurry flow to examine pipe resistance loss and wear problems. Building on these findings, Wu et al. [18] utilized cemented gangue–fly ash as a material to assess its mobility and developed a model to simulate the flow behavior of slurry in the pipeline circuit, a model that was verified by simulation. The extant studies provide a certain degree of guidance for the optimization of the filling pipeline system. However, the traditional resistance loss calculation methods principally rely on empirical formulas or numerical simulations, which are difficult to adapt to the complex and variable working conditions.

The advent of Industry 4.0 [19,20,21,22,23] has inaugurated a novel technological trajectory for the optimization of pipeline delivery systems in the context of fill mining. The rapid development of the Internet of Things (IoT) and big data analytics has enabled the integration of smart sensors capable of collecting real-time operational data of pipeline systems. Concurrently, advancements in artificial intelligence (AI) technology have facilitated the implementation of deep learning algorithms, enabling the analysis of the collected data. Peng et al. [24] redeveloped the ABAQUS model and integrated it with gradient boosting regression tree analysis to enhance the strength assessment and safety of corrosion screens for early warning of corrosion in offshore loose sandstone reservoirs. Fang et al. [25] proposed a knowledge–data co-driven model based on HCP and TLFC to improve the anti-interference and accuracy of urban flooding drainage pipe siltation diagnosis. Wan et al. [26] combined machine learning and CFD-DEM methods to study the coarse particle transport characteristics of inclined pipelines and found that DSEC is affected by the flow rate, concentration, and the inclination angle of the pipeline in a regular manner. Jiang et al. [27] proposed a robust machine learning method based on particle swarm optimization and a bi-directional gated loop unit–attention mechanism to effectively identify circulating water cooling pipeline damage in the nuclear industry. Liu et al. [28] proposed a deep reinforcement learning-based optimization framework for natural gas transportation networks, which solves problems using Markov decision processes. Compared with genetic algorithms, this framework reduces power consumption by 4.60% and saves 97.5% of the time required for dynamic programming in three typical topologies. In the context of Industry 4.0, the potential applications of IoT and AI technologies in pipeline transportation systems have been demonstrated to be significant. However, the current state of AI research on resistance loss in filled mining pipelines is suboptimal, and the exploration of related fields is in its nascent stages.

The Transformer–KAN hybrid model has demonstrated powerful modeling capabilities across multiple domains, but the focus of innovation varies across different application scenarios. In the field of virtual reality medicine, Transformer–KAN addresses the issue of error accumulation in soft tissue deformation simulation through an energy increment framework and centroid displacement time-series modeling, while leveraging Gaussian diffusion constraints to enhance global prediction accuracy [29]. In the field of geophysical logging, OMP-KAN Former [30] combines orthogonal matching tracking data augmentation with KAN modeling of nonlinear geological features and Transformer capture of long-range dependencies, significantly alleviating the challenge of scarce high-quality training data. In the field of wind power prediction, Transformer–KAN optimizes collaborative prediction of multi-source heterogeneous data through a dual-path design that separates temporal feature processing and external knowledge fusion [31]. The FlowTransKAN model developed in this paper is tailored for pipeline transportation of vertical tailings and holds advantages in industrial fluid modeling.

This paper proposes a FlowTransKAN (Flow–Transformer–KAN) network, an IoT-based multi-pressure sensor attention fusion model aimed at accurately calculating resistance losses in pipeline systems by integrating pipeline transportation experiments of tailing-filled slurry. Traditional fluid mechanics models have certain limitations in characterizing the transport properties of such non-Newtonian fluids. These models inadequately consider temperature sensitivity, and under conditions of temperature differences at underground depths in the filling system, changes in the viscosity of full tailing slurry significantly affect its flow characteristics. The abrasive effect of high-concentration slurry on pipe walls leads to a continuous increase in roughness, while traditional models using constant friction coefficients cannot reflect this time-varying characteristic, resulting in prediction errors for conveyance resistance. Traditional machine learning methods (such as decision trees and support vector machines) have been widely applied in industrial fields, but they still face challenges in different scenarios. The limited expressive capability of models makes it difficult to effectively capture complex nonlinear features and interactions in data. When faced with high-dimensional sparse data, models often exhibit poor generalization performance. Additionally, most traditional methods adopt static modeling strategies, lacking adaptive capabilities for dynamic systems, and are unable to adapt to the time-varying data distributions commonly encountered in industrial scenarios.

The main contributions of this paper are as follows:

1. By learning the distribution patterns of pipeline fluid data through a flow-based model and combining generative artificial intelligence technology to generate augmented data that conforms to historical patterns, this paper effectively addresses the limitations of traditional methods in sparse data and dynamic environments.
2. The TransKAN hybrid network architecture is designed, combining the global modeling capabilities of the Transformer with the nonlinear fitting advantages of the KAN network. The Transformer’s self-attention mechanism effectively extracts long-term dependencies in pipeline fluid dynamics features, while the KAN replaces traditional fixed activation functions with learnable spline functions, significantly improving the processing efficiency for high-dimensional sparse data.
3. Real-time collection of multi-dimensional pipeline sensor data is achieved through IoT technology, establishing a data-driven dynamic prediction system. Spatio-temporal correlation features of pipeline pressure sequences are captured, and adaptive adjustments are made to accommodate complex and variable industrial conditions, providing a scalable technical solution for intelligent pipeline transportation systems.

The paper is structured as follows: Section 2 presents experimental investigations of tailing slurry’s rheological properties, aiming to characterize its flow behavior during pipeline transport and establish a suitable resistance loss model. The experiments determine key rheological parameters (viscosity, yield stress, and shear rate) of the slurry, providing essential data for engineering applications. Section 3 details full-scale pipeline transport experiments with tailings backfill slurry, investigating how various filling multiplicities affect slurry flow behavior and resistance losses. The experimental design encompasses a multitude of variables, and a series of pressure sensors are utilized to monitor pressure variations at various locations within the pipeline. The subsequent section, Section 4, establishes the flow-based model and TransKAN network, and the model is trained based on the pipe conveying experimental data. The experimental results are presented in Section 5, and the results are compared using multiple machine learning models. This ultimately validates the highly accurate prediction capability of the FlowTransKAN model.

## 2. Experimental Design

### 2.1. Experiments on Rheological Parameters of Tailing Sand Slurry

**Figure 1:**
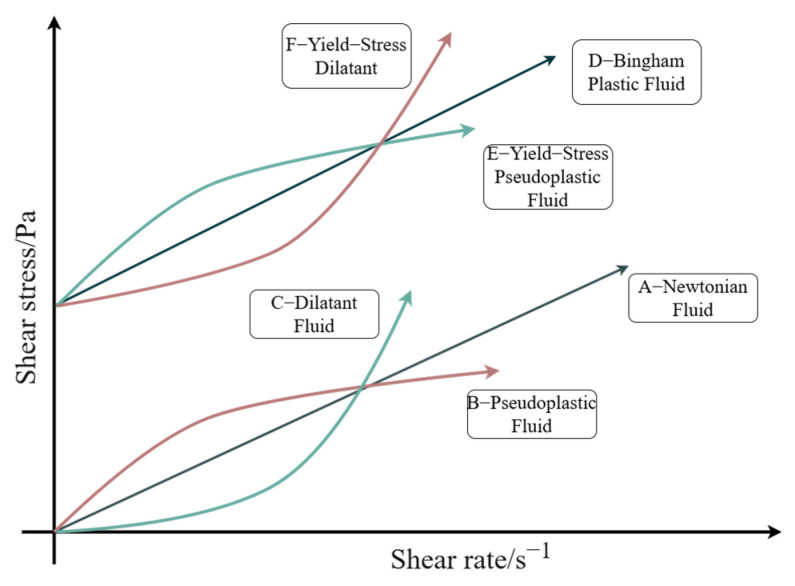
Rheological characterization of different fluids.

The rheological test of filling slurry is a pivotal method for studying the rheological properties of slurry and a key way to accurately obtain the rheological parameters [32,33,34]. Slurry flow behavior in pipeline systems demonstrates significant complexity due to multiple interacting factors, particularly under field conditions. This inherent complexity, compounded by large-scale operational variability, presents substantial challenges for comprehensive control and resistance reduction. In laboratory settings, rheometers (Thermo Fisher Scientific, MA, USA) enable precise characterization of slurry rheological properties, including viscosity, yield stress, and shear rate. Excluding time-dependent effects, the slurry’s rheological behavior can be categorized into six distinct regimes, as shown in Figure 1. Each category corresponds to a distinct flow state and a specific parameter change rule.

This study employed a rotational rheometer to characterize the rheological properties of tailing slurry, utilizing a concentric cylinder geometry with a rotating inner cylinder to shear the sample. The slurry’s viscous properties generated shear resistance against the rotating cylinder, inducing measurable changes in rotational velocity and torque. These parameters were precisely monitored in real time via integrated high-resolution sensors. The acquired signals were converted to a digital format and transmitted to a computational system, which automatically derived and displayed key rheological parameters (shear rate, shear stress, and viscosity). In this experiment, the range of shear rate was set to be 0~120 $s^{-1}$, and the measurement process lasted for 300 s to ensure the comprehensiveness and stability of data acquisition. The experimental design utilized an orthogonal test matrix to systematically evaluate key rheological determinants: mass concentration, ash-to-sand ratio, and temperature. Each variable was assigned five levels, and the specific experimental design is outlined in Table 1.

### 2.2. Experimental Study of Tailing Sand Filling Pipeline Transportation Based on IOT

**Figure 2:**
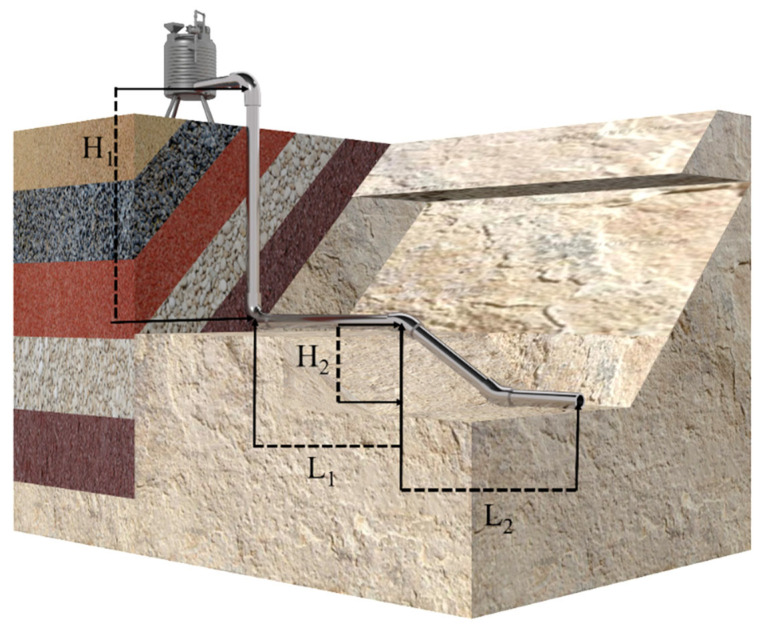
Schematic diagram of filling multiplier calculation.

Tailings pipeline transport predominantly operates in self-flow mode, where the filling multiplier serves as a key parameter defining both the self-flow capacity and operational range. This critical parameter directly reflects the engineering characteristics of mine backfill systems. Precise determination of the filling multiplier is essential for evaluating self-flow feasibility, enabling optimization of slurry transport to ensure both operational stability and cost-effectiveness. Such optimization ultimately enhances the overall efficiency and safety of mine backfill operations. The calculation of the filling multiplier is illustrated in Figure 2.

The formula for calculating the multiplication line is shown in Equation (1):

(1) $$N=\frac{L}{H}$$

where N is the filling time line, H is the filling pipeline starting point and end point of the height difference, and L includes the elbows and other pipe fittings, including the conversion length of the total length of the pipeline. It is imperative to note that the pipeline self-flow conveying filling time line should not exceed six. When the filling multiplier exceeds optimal values, mitigation strategies such as slurry concentration reduction or switching to pressurized conveying become necessary. This experiment systematically investigated four filling multipliers (3, 4, 5, and 6) under self-flow conditions to comprehensively analyze their effects on slurry hydrodynamics and resistance loss characteristics.

**Figure 3:**
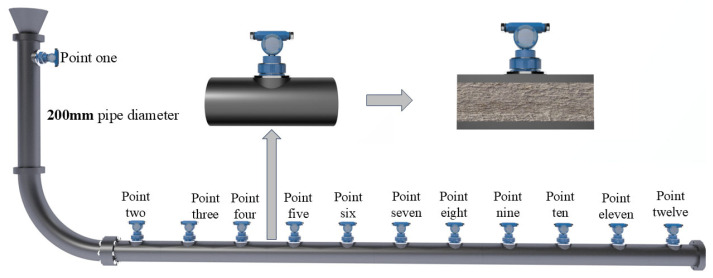
Pipeline system pressure sensor arrangement diagram.

The pipeline transmission system is the main body of the whole device, and some experimental devices are shown in Table 2. The prepared slurry is pumped to the storage tank of the upper operating platform using a mud pump. When the upper storage tank reaches the predetermined liquid level height, the valve below is opened to allow the slurry to fall freely into the vertical pipe section. A sensor point is set at the entrance of the vertical pipe section, serving as the entrance of the experimental pipeline, named the “No. 1” sensor point. At this moment, the pumping cannot stop. The upper storage tank is equipped with an overflow pipe, ensuring that the liquid level of the upper storage tank remains unchanged, thereby maintaining constant upper hydraulic pressure. The slurry flows through the elbow into the straight pipe section, where a sensor point is positioned every 50 cm, totaling 11 collection and monitoring points, as shown in Figure 3. Beginning from the elbow to the outlet of the straight pipe, they are named sensor points “2” to “12”. The slurry flows out from the outlet of the straight pipe section to the storage tank below to complete a cycle.

**Figure 4:**
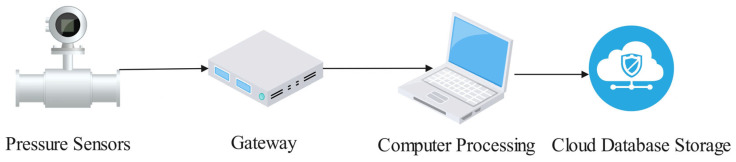
IOT-based data acquisition module.

The data acquisition system forms the experimental terminus, providing precise raw datasets for subsequent resistance analysis and modeling. As shown in Figure 4, the system comprises two core components: (1) the signal acquisition module and (2) the data storage module. The incorporation of IoT technology optimizes data transmission efficiency and reliability during acquisition. Collected signals are converted into standardized protocols via the IoT gateway before being transmitted to the local server for preliminary processing. Furthermore, the system implements real-time cloud database synchronization, enabling secure data archiving and cloud-based management. This cloud platform architecture supports efficient experimental data storage and analytical processing while providing remote monitoring capabilities for timely access to experimental status and analytical results. This capability supports informed decision-making processes. In the context of Industry 4.0, this data acquisition system not only facilitates intelligent equipment and production environment monitoring, but also provides a data foundation for the optimization and upgrading of industrial production.

In this study, a three-factor, four-level experimental scheme was designed to characterize the resistance loss in the pipeline transportation of tailing sand slurry. The mass concentration, pipe diameter, and filling multiplier were taken as the main experimental variables to systematically investigate the influence of each factor on the resistance loss. The mass concentration was set at 71%, 72%, 73%, and 74% to reflect the viscosity change of slurry under different solid–liquid ratios and its influence on the flow resistance. The pipe diameters were selected as 100 mm, 150 mm, 200 mm, and 250 mm to encompass the standard dimensions of the filling pipe, thereby facilitating a comparative analysis of the adjusting effect of the pipe diameters on the resistance loss. The filling multiplier was set at 3, 4, 5, and 6 to account for the self-flowing nature of the pipe, which is the most prevalent pipe size. The filling multipliers were set to 3, 4, 5, and 6 to consider their influence on the flow characteristics of the slurry in the pipe as an important index of the self-flow conveying capacity and filling range. The established testing system was used to conduct 64 sets of pipeline transportation tests. These tests comprehensively covered working conditions under different combinations of factors to ensure the scientific nature and representativeness of the test results. All experimental data was collected from actual tests using this experimental equipment, including key parameters such as pressure and temperature, to ensure the accuracy and completeness of the data to support the subsequent analysis and conclusions.

## 3. The Construction of Models

### 3.1. Flow-Based Model

**Figure 5:**
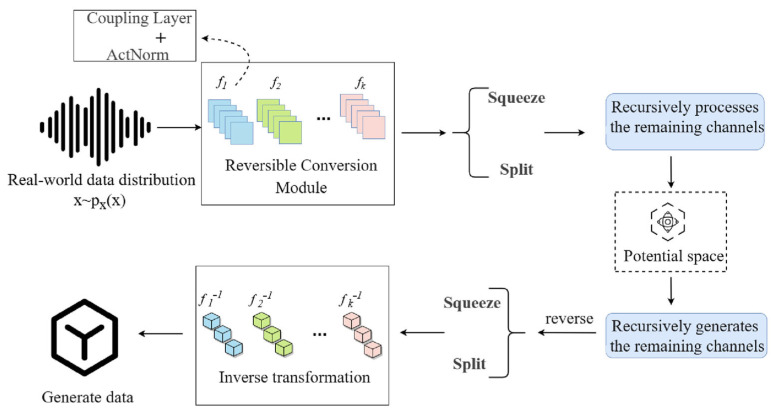
Flow-based model structure diagram.

Accurate calculation of resistance loss in full-scale tailing slurry pipelines conventionally requires extensive experimental data. However, practical constraints make comprehensive testing across multiple slurry ratios, filling multiples, and pipe diameters prohibitively expensive and time-intensive. Additionally, the acquisition of pressure data at various measurement points during testing can be challenging, and the data obtained by sensors may contain errors, further complicating data processing and analysis. These challenges hinder the development of highly accurate drag loss prediction models and underscore the need for cost-effective and time-efficient experimental methodologies that maintain data precision. In this paper, we propose a methodology that employs generative artificial intelligence to address these challenges. Specifically, we use a flow-based model to learn the data characteristics of the tailings backfill slurry pipeline, as shown in Figure 5, to construct high-quality training samples that conform to the true distribution, effectively overcoming the limitations of traditional data acquisition. However, the current generation process primarily relies on the statistical characteristics of measured data without explicitly incorporating fluid mechanics conservation laws, such as the continuity equation and Bernoulli’s equation, as hard constraints. The focus is on verifying the feasibility of generative AI in predicting pipeline resistance losses in mining applications, with a core emphasis on uncovering intrinsic data correlations to avoid model performance limitations caused by assumption biases.

The reasoning of the flow-based model is predicated on the variable substitution formula. Let the invertible transformation be denoted by $z_{i}=f_{\theta_{i}}(z_{i-1})$, where $\theta_{i}$ is the set of parameters of the i-step transformation. According to the established mathematical framework, the invertible transformation, $f_{\theta_{i}}$, possesses the property that there exists a unique inverse transformation, $f_{\theta_{i}}^{-1}$, satisfying the following condition:

(2) $$f_{\theta_{i}}^{-1}(f_{\theta_{i}}(z_{i-1}))=z_{i-1}$$

This guarantees that information is not lost during the transformation process. According to the variable substitution formula, the probability density functions $p_{z_{i-1}}(z_{i-1})$ and $p_{z_{i}}(z_{i})$ of random variables $z_{i-1}$ and $z_{i}$ are related as follows:

(3) $$p_{z_{i}}(z_{i})=p_{z_{i-1}}(z_{i-1})\left|\det\left(\frac{\partial f_{\theta_{i}}^{-1}}{\partial z_{i}}\right)\right|$$

In this equation, $\det\left(\frac{\partial f_{\theta_{i}}^{-1}}{\partial z_{i}}\right)$ denotes the Jacobi determinant. Due to the property of invertible transformation, $\frac{\partial f_{\theta_{i}}^{-1}}{\partial z_{i}}={\left(\frac{\partial f_{\theta_{i}}}{\partial z_{i-1}}\right)}^{-1}$, the equation can be further rewritten as follows.

(4) $$p_{z_{i}}(z_{i})=p_{z_{i-1}}(z_{i-1})\left|{\left[\det(\frac{\partial f_{\theta_{i}}}{\partial z_{i-1}})\right]}^{-1}\right|$$

The probability density, $p_{x}(x)$, of x can be derived recursively from the probability density of $z_{0}$ when starting from a random variable, $z_{0}$, that initially obeys a simple distribution (usually the standard normal distribution $p_{z_{0}}(z_{0})$) and undergoes a K-step invertible transformation that eventually yields a variable, x, i.e., $x=f_{\theta_{K}}(\cdots f_{\theta_{2}}(f_{\theta_{1}}(z_{0}))\cdots )$; the mathematical expression for the probability density $p_{x}(x)$ of x is as follows. The specific mathematical expression is

(5) $$p_{x}(x)=p_{z_{0}}(z_{0})\prod_{i=1}^{K}\left|{\left[\det(\frac{\partial f_{\theta_{i}}}{\partial z_{i-1}})\right]}^{-1}\right|$$

where $z_{0}=f_{\theta_{1}}^{-1}(\cdots f_{\theta_{K-1}}^{-1}(f_{\theta_{K}}^{-1}(x))\cdots )$.

In the training phase of the model, the maximum likelihood estimation method is typically employed by the flow-based model. For a given training dataset, ${\left\{x^{\left(n\right)}\right\}}_{n=1}^{N}$, its log-likelihood function is defined as

(6) $$L(\theta )=\frac{1}{N}\sum_{n=1}^{N}\log p_{x}(x^{\left(n\right)})$$

The fundamental principle of maximum likelihood estimation is to maximize the probability of the model generating training data by finding an optimal set of parameters, θ, which maximizes the probability of the model generating training data. Substituting the previously derived expression for $p_{x}(x)$ into the log-likelihood function yields

(7) $$L(\theta )=\frac{1}{N}\sum_{n=1}^{N}\left(\log p_{z_{0}}(z_{0}^{\left(n\right)})-\sum_{i=1}^{K}\log\left|\det(\frac{\partial f_{\theta_{i}}}{\partial z_{i-1}^{\left(n\right)}})\right|\right)$$

where $z_{0}^{\left(n\right)}$ is obtained from $x^{\left(n\right)}$ by inverse transformation. During the training process, the value of the log-likelihood function, denoted by L(θ), is maximized by continuously optimizing the parameter $\theta =(\theta_{1},\theta_{2},\ldots ,\theta_{K})$. This optimization process is typically achieved with the aid of optimization algorithms, such as gradient descent. This process involves calculating the gradient of the log-likelihood function with respect to the parameters and gradually adjusting the parameter values in the direction of the gradient. This adjustment enables the model to better fit the distribution of the training data. This process enables the model to discern intricate patterns and statistical characteristics inherent in the data, thereby facilitating the generation of high-quality samples.

### 3.2. Establishment of the KAN Network

**Figure 6:**
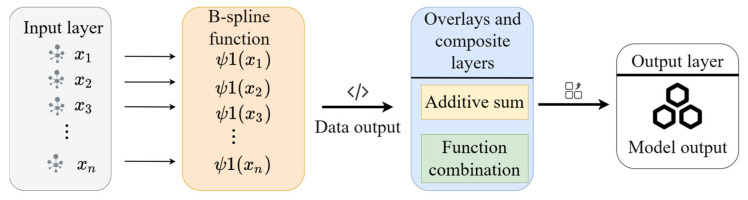
KAN network structure diagram.

The KAN network is a method that combines neural networks and B-splines to efficiently fit complex nonlinear relationships in data, as illustrated in Figure 6. This figure presents a brief structure of the KAN network. The KAN network enhances the expressive power of the network by introducing the B-spline function to process the input data, especially in the case of high-dimensional data and complex tasks. The application of the KAN network to the calculation of resistance loss in the pipeline transportation process aims to solve the problem of resistance loss calculation when tailing slurry is transported in a pipeline. Conventional pipeline resistance loss calculation methods generally depend on experimental data. However, data collection is frequently arduous and time-consuming. The integration of the B-spline function within the KAN network facilitates the effective modeling of complex nonlinear relationships and enhances the prediction accuracy of high-dimensional data. The KAN network’s capacity to circumvent the overfitting phenomenon in high-dimensional data settings is attributable to its incorporation of the localization, non-negativity, and additivity characteristics inherent in the B-spline basis function. Concurrently, the KAN network enhances the efficacy of processing complex pipeline pressure fluid data. In certain applications, the KAN network is trained using rheological experiments and pipelines conveying experimental data. This training enables the network to accurately predict resistance loss in the pipeline. It also optimizes the design of the filling system and enhances the overall efficiency and safety of the mine filling project.

**Figure 7:**
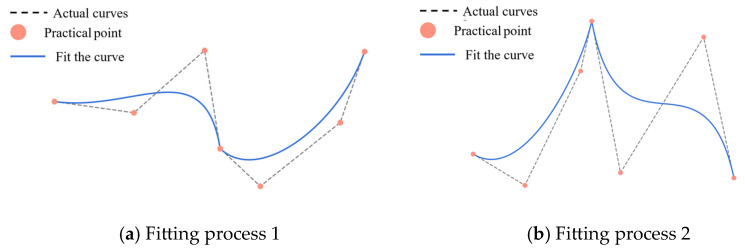
Cubic spline interpolation schematic.

The B-spline function is a widely utilized tool in interpolation and fitting, employed to delineate a smooth function curve through a set of control points, as illustrated in Figure 7 for the cubic spline difference. The B-spline basis function is distinguished by local support, implying that each basis function is non-zero only in a finite interval. This ensures that the basis function is localized and does not extend beyond the boundaries of the curve. Secondly, the B-spline basis function is non-negative, meaning that the value of each basis function is always non-negative. This property ensures the reasonableness of the calculation results. Finally, the B-spline basis function has additivity, i.e., the sum of all the basic functions is always 1. This property enables the B-spline to maintain smoothness during the interpolation process and avoids the occurrence of the overfitting phenomenon. The B-spline basis function, $N_{i,p}(t)$, depends on the distribution of the nodes as well as the order, p, of the spline function, and the zero-order spline basis function is

(8) $$N_{i,0}(t)=\left\{\begin{array}{l} 1\\ 0 \end{array}\right.\begin{array}{c} \mathrm{if}\;t_{i}\leq t<t_{i+1},\\ \mathrm{otherwise}. \end{array}$$

For p>0, the B-spline basis function $N_{i,p}\left(t\right)$ is defined by the following recursive relationship:

(9) $$N_{i,p}(t)=\frac{t-t_{i}}{t_{i+p}-t_{i}}N_{i,p-1}(t)+\frac{t_{i+p+1}-t}{t_{i+p+1}-t_{i+1}}N_{i+1,p-1}(t)$$

The KAN network is predicated on a B-spline function that draws inspiration from the traditional neural network. Each KAN layer is comprised of two components: a standard linear transform component and a B-spline transform component. The input to the KAN layer is a batch of data of size $\left(B,d\right)$, where B denotes the batch size and d signifies the dimension of the input features. The computation of the KAN layer is constituted of two components: a linear transform component and a B-spline transform component.

The output of the linear transformation component is derived by multiplying the input, x, by the weight matrix, W, and adding the bias, b.

(10) LinearOutput=Wx+b

The weight matrix $W\in {\mathbb{R}}^{d^{\prime }\times d}$, and the output dimension of the linear transformation is $d^{\prime }$.

The B-spline transform part is the processing of the input, x, by a B-spline basis function. Assume that the input, x, is mapped to the nodes of the B-spline function and that a smooth output is generated by the B-spline basis function. Suppose that the output of the B-spline transformation is

(11) $$\text{B-Spline}(x)=\overset{d^{\prime }}{\underset{i=1}{\sum }}N_{i,p}(x)$$

where $N_{i,p}(x)$ is the B-spline basis function, p is the order of the B-spline, and $d^{\prime }$ is the output dimension.

Combining the above, the KAN layer is calculated as

(12) y=Wx+b+B-Spline(x)

In the KAN layer, the B-spline Transform component serves to smooth the input data and enhance the nonlinear fit of the model.

The KAN network is structured with multiple layers, with the output of each layer serving as the input to the next. This hierarchical structure enables the KAN network to learn high-level features of the data layer by layer. The architecture of the KAN network is defined by L layers, with the input of each layer designated by $x_{l}$ (where l denotes the number of layers). The output of the lth layer is then defined as

(13) $$x_{l+1}={\mathrm{KAN}}_{l}(x_{l})$$

where ${\mathrm{KAN}}_{l}(x_{l})$ denotes the computational process of the lth layer in the KAN, comprising both linear transformation and B-spline transformation.

The KAN network significantly improves neural networks’ nonlinear fitting capacity through B-spline basis function integration while employing smoothing techniques to mitigate overfitting. Each KAN layer’s computational architecture combines linear transformations with B-spline operations, enabling superior performance in high-dimensional data processing and complex task execution.

### 3.3. FlowTransKAN Modeling

**Figure 8:**
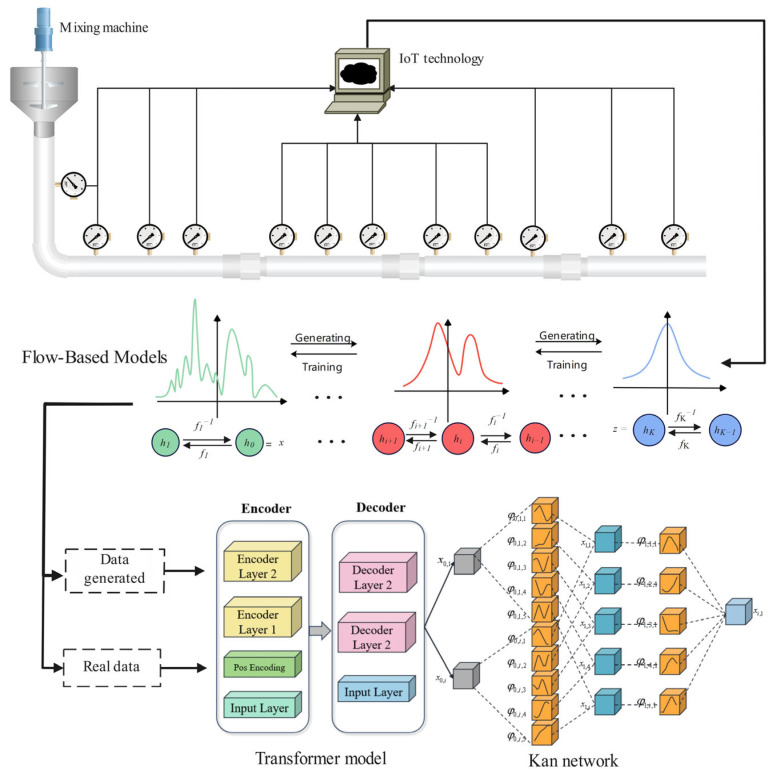
FlowTransKAN model structure diagram.

The Transformer [35,36] is predicated entirely on the self-attention mechanism and the multi-head attention mechanism, thus distinguishing it from traditional recurrent neural networks and long- and short-term memory networks. This configuration facilitates the effective capture of global dependencies in the input data through parallel computation. The Transformer module is integrated into the KAN network to establish the TransKAN model, as depicted in Figure 8. The Transformer module has the capacity to establish long-distance dependencies between various parts of the input data, and the self-attention mechanism is able to dynamically assign a long-distance dependency relationship to each part of the input data in the pipeline pressure resistance loss task. The attention mechanism enables the dynamic allocation of different weights to different parts of the input data, thereby more accurately reflecting the influence relationship between different points. The KAN network enhances the network’s expressive ability, enabling higher-order nonlinear fitting and improving the overall prediction effect.

By combining the flow-based model with the TransKAN network, the flow-based model implicitly incorporates fluid mechanics constraints through a data-driven approach to generate augmented data that conforms to the real distribution, while TransKAN captures complex spatio-temporal features through attention mechanisms and nonlinear fitting, ultimately achieving high-precision prediction of pipeline resistance loss; the pseudo-code of the FlowTransKAN algorithm is Algorithm 1.
**Algorithm 1.** FlowTransKAN1: Input: Pipeline pressure data X = {x_1_, x_2_, …, x_T} 2: Output: Predicted value y 3: procedure FLOW_BASED_MODEL() 4:      z = sample ()//Sample latent variable from normal distribution 5:      X_fake = z 6:      for t from T down to 1 do 7:           (scale, shift) = NeuralNet(X_fake)//Predict scale and shift parameters 8:           X_fake = (X_fake − shift)/scale//Inverse process: generate data from latent space 9:      end for 10:     return X_fake 11: end procedure 12: procedure TRANSFORMER(X) 13:      A = MultiHeadSelfAttention(Q = X, K = X, V = X)//Compute multi-head self-attention 14:      A_res = LayerNorm(X + A)//Add residual connection and layer normalization 15:      return A_res 16: end procedure 17: procedure KERNEL_ATTENTION_NETWORK(A_res) 18:      H = zeros_like(A_res)//Initialize output matrix 19:      for t from 1 to T do
20:           for q in 1 to Q do
$21:\;\;\;\;\;\;\;\;\;\;\;\;\;\;\;\;z_{q}=\sum_{p=1}^{d_{model}}\phi_{q,p}(A_{res}[t,p])$//Compute scalar projection for each basis function group 22:           end for
$23:\;\;\;\;\;\;\;\;\;\;\;h_{t}=\sum_{q=1}^{Q}\;\Phi_{q}(z_{q})$//Aggregate results 24:          H[t] = H[t] + h_t//Update output matrix 25:      end for 26:      H_out = LayerNorm(A_res + H)//Apply residual and layer normalization 27:      return H_out 28: end procedure

**Figure 9:**
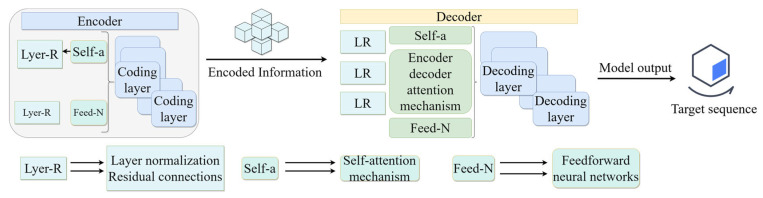
Schematic structure of Transformer model.

The Transformer model is composed of two primary components: the encoder and the decoder, as illustrated in Figure 9. Information flow between the encoder and decoder is mediated through a self-attention mechanism and feed-forward network. The Transformer’s key advantage lies in its parallel processing architecture, which simultaneously analyzes all sequence elements while effectively capturing long-range dependencies.

The self-attention mechanism constitutes the core technology of the Transformer, a sophisticated machine learning model capable of computing the relationship between each element and other elements in a sequence to obtain global information. For each element in the input, the self-attention mechanism generates a weighted representation by computing the relationships between queries, keys, and values.

Given input data $X=[x_{1},x_{2},\ldots ,x_{T}]$, where T is the sequence length and $x_{i}\in {\mathbb{R}}^{d}$ is the feature vector of the ith element, each input element, $x_{i}$, is mapped to three distinct vector spaces: Query, Q; Key, K; and Value, V. Specifically, the input, $x_{i}$, is transformed into queries, keys, and values via learned weight matrices:

(14) $$Q_{i}=x_{i}W_{Q},K_{i}=x_{i}W_{K},V_{i}=x_{i}W_{V}$$

In the above equation, $W_{Q}$, $W_{K}$, and $W_{V}$ are the weight matrices obtained from learning; d is the feature dimension; $d_{Q}$, $d_{k}$ and $d_{v}$ are the query, key and value dimensions, respectively.

To compute the similarity between each query, Q, and all keys, K, we compute the dot product of the query and the keys and normalize it with the Softmax function:

(15) $$\mathrm{Attention}(Q,K,V)=\mathrm{softmax}\left(\frac{QK^{T}}{\sqrt{d_{k}}}\right)V$$

where $\frac{1}{\sqrt{d_{k}}}$ is the scaling factor to prevent the dot product from being too large and causing the gradient to disappear or explode. The attentional weights between each query and all keys are obtained after normalization by the Softmax function. These attentional weights are multiplied by the value V to obtain the weighted output.

The Transformer computes different attention scores in parallel through multiple self-attentive heads, thereby enabling the model to understand the input data from multiple perspectives. Each head possesses independent query, key, and value weight matrices. Ultimately, the outputs of all the heads are integrated, and a linear transformation is applied to obtain the final output.

(16) $$\mathrm{MultiHead}(Q,K,V)=\mathrm{Concat}({\mathrm{head}}_{1},{\mathrm{head}}_{2},\ldots ,{\mathrm{head}}_{h})W_{0}$$

The computation of each header is outlined as follows:

(17) $${\mathrm{head}}_{i}=\mathrm{Attention}(Q^{\left(i\right)},K^{\left(i\right)},V^{\left(i\right)})$$

where $\;W_{0}$ is the linear transformation matrix of the output, and the spliced output will contain information from multiple subspaces.

The Transformer model, devoid of a recursive structure, is deficient in a mechanism to process sequential information. In the context of time-series data, the order of the time steps is paramount, necessitating the incorporation of positional information through position encoding. Position encoding is computed by sine and cosine functions and incorporated into the feature representation of the input data, thereby providing positional information for each element. The formula for position encoding is as follows:

(18) $$PE(t,2i)=\sin\left(\frac{t}{10,000^{2i/d}}\right),PE(t,2i+1)=\cos\left(\frac{t}{10,000^{2i/d}}\right)$$

where t is the position, i is the dimension index, and d is the dimension of the position encoding. The position encoding is added to the embedded:

(19) $$X_{\mathrm{pos}}=X+PE$$

Each layer of the Transformer encoder and decoder is followed by a feed-forward neural network, which is used to perform a nonlinear transformation on each element. Feed-forward neural networks typically contain two fully connected layers and use the ReLU activation function.

(20) $$FFN(x)=\max(0,xW_{1}+b_{1})W_{2}+b_{2}$$

where $W_{1}$, $W_{2}$ is the weight matrix; $b_{1}$, $b_{2}$ is the bias; and the ReLU activation function is used to increase the nonlinearity.

## 4. Results

### 4.1. Experimental Results on Rheological Parameters of Tailing Sand Slurries

**Figure 10:**
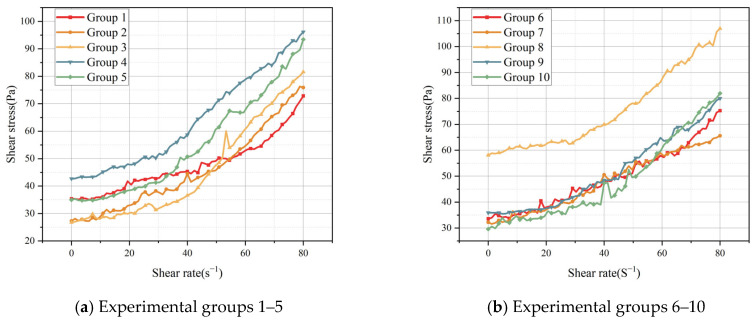
Rheological experimental diagram for tailing sand slurry.

The rheological experiments on the tailing sand slurry, which employed a rheological apparatus, yielded the following results, as illustrated in Figure 10. The observed fluid behavior aligns more closely with the Bingham-type flow characteristics. The Bingham-type flow is distinguished by the presence of yield stress, which denotes that the fluid begins to flow only when the applied stress surpasses a critical threshold (yield stress). Consequently, under conditions of low stress, the fluid manifests the properties of a solid and is incapable of flow, only beginning to deform when sufficient external force is applied.

(21) $$\tau =\tau_{0}+\eta \left(\frac{d_{u}}{d_{t}}\right)$$

For the study of tailing slurry, the experimental finding that its flow characteristics conform to the Bingham-type flow-based model is of great significance. The tailing slurry is composed of solid particles and liquid, and its flow characteristics are affected by factors such as particle size, concentration, shape, and distribution. Through experimental studies, it was found that tailing slurry exhibits typical Bingham-type flow characteristics, which can better describe the behavior of non-Newtonian fluids containing larger particles. The yielding phenomenon of the fluid when stress is applied reflects the interaction between the particles and the structural strength, which reveals the flow characteristics and critical flow conditions of the tailing sand slurry under specific stress.

The conveying, handling, and storage of tailing slurries in mine filling processes generally necessitate the overcoming of elevated yield stresses. The rheological parameters obtained from experimental studies can assist in optimizing the design and operating conditions of the equipment. This, in turn, can reduce adverse phenomena such as clogging and sedimentation that may occur during the flow process. Consequently, this can improve the operating efficiency and reduce energy consumption and costs. The Bingham-type flow characteristics of the tailing slurry facilitate a more profound comprehension of the underlying flow mechanisms and provide a theoretical foundation for the enhancement of pertinent engineering technologies, thereby ensuring the stability and reliability of the tailing slurry treatment process.

Experiments have been conducted to determine the rheological parameters, yielding the yield stress and plastic viscosity. In accordance with the theory of hydrostatic equilibrium, the pressure difference between the two ends of a straight pipe of length  l is equivalent to the frictional resistance of its inner wall. This relationship is expressed in Equations (22) and (23).

(22) $$0.25\pi D^{2}\times ∆P=\pi D\times \tau_{B}\times l$$

(23) $$i=\frac{4\tau_{B}}{D}$$

In the formula, D denotes the pipe diameter, l denotes the length, and $\tau_{B}$ denotes the shear stress. The average flow rate of the filling slurry under pipe transport conditions can be derived from Buckingham’s formula, as demonstrated in the following Equation (24):

(24) $$v=\frac{\tau_{g}D}{8\mu }\left[1-\frac{4}{3}\left(\frac{\tau_{0}}{\tau_{B}}\right)+\frac{1}{3}{\left(\frac{\tau_{0}}{\tau_{B}}\right)}^{4}\right]$$

In the above formula, $\tau_{0}$ represents the yield stress; μ represents the plastic viscosity, for the concentration of larger filling slurry in the tube, because of its faster movement, can be considered a high concentration of filling slurry, such that the yield stress is much smaller than the shear stress; and $\frac{\tau_{0}}{\tau_{B}}$ of the higher power can be approximated as zero, such that it can be ignored here, so Equations (23) and (24) can be obtained by the Buckingham formula for the association:

(25) $$i=\frac{16}{3D}\tau_{0}+\frac{32v}{D^{2}}\mu$$

Equation (25) in i represents the unit length along the resistance loss for the calculation of pipeline resistance loss of the basic model. In order to obtain the pipeline resistance loss, a large number of experiments on the rheological parameters must be carried out. Assumptions are made during the calculation process, and the calculated values may differ significantly under different working conditions.

### 4.2. Test Results of Pipeline Transport of Full Tailing Sand Fill Slurry

**Figure 11:**
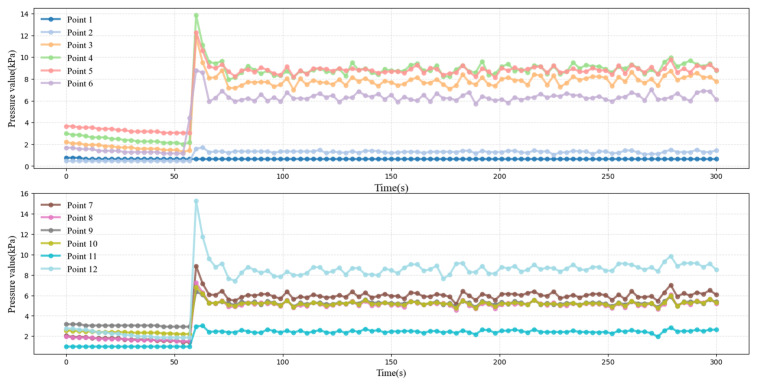
Full tailing sand filling slurry pipeline conveying pipeline pressure chart.

In the tailing sand filling slurry pipeline transport test, the measurement time for each set of experiments was 300 s. To minimize the influence of random errors on the experimental results, each set of experiments was repeated three times. During each experiment, data from 12 pressure sensors were collected in real time to monitor pressure changes at various locations in the pipeline, as shown in Figure 11. This data provides a sufficient basis for a comprehensive understanding of the pressure distribution characteristics of the slurry inside the pipe, which can reflect the detailed changes in the slurry flow process. In addition, the data collected provides important support for subsequent rheological analysis and model validation.

Through the process of visualizing and analyzing a segment of the experimental data, it was determined that point 1 was situated within the vertical pipe section, where the pressure levels were minimal and the fluctuation patterns were deemed to be unstable. Consequently, these anomalous data points were identified and subsequently removed during the data preprocessing stage. Subsequent analysis revealed that the data fluctuated less during the time from 50 to 300 s and exhibited a more stable trend, which was consistent with the normal change rule under the experimental conditions. To ensure the accuracy and representativeness of the subsequent data analysis, the data from this time was selected for the calculation of the resistance loss, thereby avoiding the unnecessary influence of unstable data on the results.

**Figure 12:**
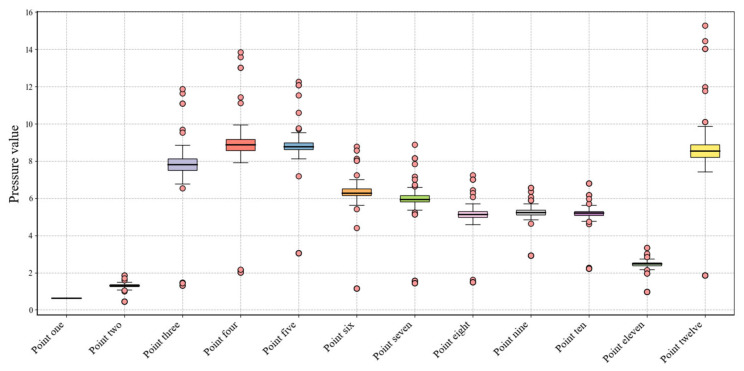
Data preprocessing box–line diagram.

To minimize random error interference, we performed a boxplot analysis (Figure 12) to identify and exclude potential outliers from each experimental dataset. This data preprocessing step significantly improved measurement accuracy and result reliability while eliminating extreme value effects, thereby enhancing the scientific rigor of the subsequent analyses.

### 4.3. Predicted Results of Pipeline Resistance Losses

In hydrodynamic studies of piping systems, pipe resistance loss is an important measure of the work required for fluid flow. For both datasets, we used several input features to predict the resistance loss of the pipeline, including variables such as mass concentration, ambient temperature, gray-to-sand ratio, and multiplier line. These variables can have a significant impact on pressure changes during pipeline delivery, and accurately modeling the relationship between these factors and pressure is key to predicting pipeline resistance losses. A machine learning model was used to predict the pressure values at each point in time or at each measurement point, and to obtain the final value of the pipeline resistance loss, the pressure difference between each two consecutive measurement points was summed to obtain the resistance loss of the pipeline system during the entire process. There were N pressure measurement points in the pipeline, and the pressure values at these points were recorded as $P_{1},P_{2},P_{3},\ldots ,P_{N}$, where $P_{i}$ is the pressure at the ith measurement point.

For two neighboring points, i and i+1, the pressure difference between them is expressed as

(26) $$∆P_{i}=P_{i+1}-P_{i}$$

In this equation, $∆P_{i}$ denotes the pressure difference between points i and i+1. The total resistance loss, denoted by $P_{\mathrm{loss}}=∆P_{\mathrm{total}}$, is calculated by accumulating the pressure difference between all neighboring points:

(27) $$∆P_{\mathrm{total}}=\overset{N-1}{\underset{i=1}{\sum }}(P_{i+1}-P_{i})$$

In order to provide a basis for comparison with the model constructed in this paper, several common models were employed.

BP neural network: This is a multi-layer feed-forward neural network that learns the nonlinear relationship between the input and the output by adjusting the weights through a back-propagation algorithm. Typically, it comprises an input layer, a hidden layer, and an output layer, with the objective of enhancing performance through minimizing error.

Support vector machine (SVM): This is a supervised learning method for classification and regression. It performs data segmentation by finding the optimal hyperplane in a high-dimensional space and can handle both linear and nonlinear problems and improves the nonlinear fitting ability of the model through the kernel function.

Random forest: This integrated learning method enhances prediction accuracy by constructing numerous independent decision trees and by implementing voting or averaging mechanisms.

Two datasets were established: the original experimental data and the dataset after expansion using the flow-based model. The experimental data was divided into a training set, a test set, and a validation set at a ratio of 8:1:1. The $R^{2}$, RMSE, and MAE were used to evaluate the hybrid model. Each evaluation criterion was calculated as follows:

(28) $$R^{2}=1-\frac{\sum_{i=1}^{n}{\left(y_{i}-{\hat{y}}_{i}\right)}^{2}}{\sum_{i=1}^{n}{\left(y_{i}-\overset{–}{y}\right)}^{2}}$$

(29) $$\mathrm{RMSE}=\sqrt{\frac{1}{n}\overset{n}{\underset{i=1}{\sum }}{\left(x_{i}-{\hat{x}}_{i}\right)}^{2}}$$

(30) $$\mathrm{MAE}=\frac{1}{n}\overset{n}{\underset{i=1}{\sum }}|x_{i}-{\hat{x}}_{i}|$$

In this equation, n denotes the number of samples, $x_{i}$ signifies the measured value of the ith sample, ${\hat{x}}_{i}$ represents the predicted value of the ith sample, $\bar{x}$ constitutes the average value of the samples, $y_{i}$ is the actual observed value, ${\hat{y}}_{i}$ is the model predicted value, $\bar{y}$ is the average of the observations, and n is the number of samples.

### 4.4. Ablation Experiment

To verify the effectiveness of each module in the FlowTransKAN model, an experimental design was developed to conduct a comprehensive analysis of its contribution to the overall performance. The experimental design utilized R^2^, MAE, and RMSE as evaluation metrics. The ensuing experimental results are delineated in Table 3.

**Figure 13:**
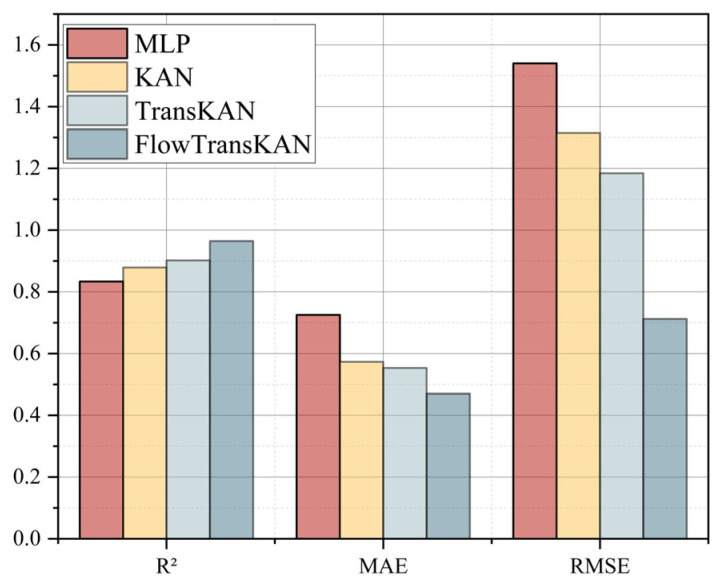
Histogram of ablation experiment results.

In the following ablation experiments, the effectiveness of the models was verified by analyzing the performance differences between KAN, TransKAN, and FlowTransKAN and using the neural network MLP as a reference benchmark. As illustrated in Figure 13, the KAN model demonstrates superior performance in terms of R^2^, MAE, and RMSE metrics when compared to the MLP model. An analysis of the underlying logic of the MLP and the KAN models reveals that the MLP’s deficiency stems from its reliance on a substantial number of neurons to compensate for the constrained expressive capacity of the fixed activation function. In contrast, the KAN model replaces the fixed activation function with a learnable spline function, thereby enhancing the approximation efficiency. This renders the KAN model more suitable for tasks that demand precise representation of intricate mathematical relationships. A comparison of TransKAN and KAN reveals that TransKAN outperforms KAN in terms of the RMSE index and demonstrates higher stability in prediction results. Additionally, TransKAN exhibits superior performance in terms of R^2^ and MAE. This enhancement can be attributed to the incorporation of the self-attention mechanism of the Transformer into the KAN model. The Transformer mechanism enhances the model’s capacity to acquire global data and discern patterns, thereby improving its predictive accuracy. Consequently, it can be concluded that the incorporation of a self-attention mechanism within a Transformer framework enhances the stability of the model’s prediction outcomes. The experimental results demonstrate that the prediction stability of FlowTransKAN is significantly superior to that of TransKAN, with RMSE values of 0.7126 and 1.1841, respectively, indicating a relative reduction of 39.8%. This discrepancy signifies that the Flow algorithm enhances the model’s resilience through parameter optimization. Specifically, Flow maximizes the log-likelihood function through gradient descent during training, thereby driving the model to accurately fit the data distribution. This optimization not only enhances the model’s ability to capture complex statistical features but also systematically improves stability by constraining the continuity of the output space and reducing the sensitivity of the prediction results to input perturbations.

The integration of the Flow, KAN, and Transformer models in the FlowTransKAN framework significantly improves prediction stability and accuracy. This hybrid architecture combines their respective strengths to deliver optimal performance for industrial applications, enabling precise scientific predictions of slurry pipeline resistance loss and other processes, even with limited sample data.

### 4.5. Comparison Experiment

In the interdisciplinary field of fluid mechanics and industrial engineering, the application of machine learning algorithms in predicting pipeline resistance loss is still in its exploratory phase. Particularly for high-concentration complex media such as tailing slurry in mine backfill systems, research and analysis on resistance loss prediction during pipeline transportation remain insufficient. Given the long-term application value of traditional mathematical models in this field, this study used mathematical models as a benchmark reference system and selected models commonly used in industrial applications for comparative experiments. The FlowTransKAN model was compared with BP neural networks, random forests, SVM models, and the Edgar Buckingham formula. The training of Flow used only new instances for training the improved model proposed in this paper to fully demonstrate the improvement of this method under the same data conditions. The final results are shown in Table 4.

A comparative analysis of training efficiency was conducted using a unified hardware environment based on the CUDA parallel computing architecture featuring the Intel Core i7-13650HX processor (Intel, SantaClara, CA, USA) and an NVIDIA RTX 3090 graphics card (NVIDIA, SantaClara, CA, USA). As shown in Table 4, the proposed FlowTransKAN model requires 450 s to complete training, which is significantly longer than traditional algorithms such as random forest (85 s) and the support vector machine (120 s) but is comparable to the back-propagation (BP) neural network (300 s). The computational overhead primarily stems from the self-attention operations in the Transformer encoder and the parameterized computation process of the KAN activation function within the model architecture. For application scenarios such as real-time monitoring of mine backfilling projects, considering that model training is an offline operation and the single prediction latency is below 50 ms, the model achieves a balance between computational resource investment and accuracy.

We systematically adjusted the parameters of all comparison models to optimize their performance. By optimizing the parameters of each model on a fair basis, we were able to more objectively demonstrate the performance advantages of the improved model proposed in this paper. Table 5 shows the key parameters of the model.

**Figure 14:**
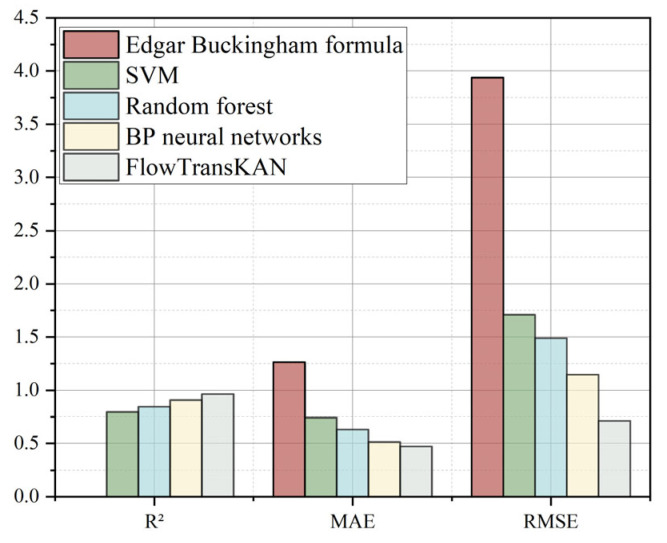
Comparison of experimental results histogram.

To quantify the discrepancies between the models, the results are presented in the form of a histogram, as shown in Figure 14. The results indicate that FlowTransKAN outperforms comparative models across all evaluation metrics. This performance enhancement stems from (1) the Transformer’s self-attention mechanism, which improves global data pattern recognition, and (2) the integrated KAN–Flow architecture, where spline-based activation replaces fixed activation functions, eliminating the need for excessive neurons to compensate for limited nonlinear representation capacity. This approach enhances approximation efficiency and reduces data acquisition costs while ensuring the model maintains high prediction accuracy even with limited samples. Although Edgar Buckingham’s formula, as a classical physical theorem, has broad applicability, it lacks the flexibility and adaptability required for complex, multi-factor coupled pipeline resistance loss problems. Similarly, support vector machines exhibit limitations in capturing intricate nonlinear relationships, potentially failing to adequately model the complex patterns inherent in pipeline resistance loss. Random forest is occasionally prone to overfitting, particularly when trained on large-scale pipeline datasets. This overfitting tendency may compromise the model’s generalization performance when applied to unseen data. While the BP neural network demonstrates strong capability in modeling complex nonlinear relationships, its performance in fitting pipeline pressure data requires further improvement. In contrast, the proposed FlowTransKAN model exhibits superior performance in capturing intricate data patterns and generating accurate predictions compared to conventional and classical approaches.

### 4.6. Sensitivity Analysis

To evaluate the model’s robustness to environmental variables, temperature fluctuations and sensor noise perturbations were introduced into the experimental data. Specifically, temperature variations were analyzed under fixed parameters. For sensor noise, Gaussian noise was added to pressure data to simulate varying levels of measurement errors.

**Figure 15:**
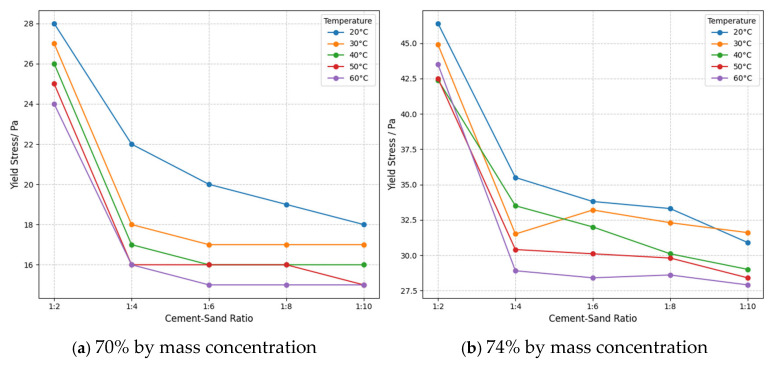
Yield stress versus sand-to-cement ratio curve.

In sensitivity analysis, simulating temperature fluctuations is a crucial aspect of evaluating model robustness to environmental variables. Experiments systematically analyzed the response characteristics of model outputs to temperature variations under fixed parameter conditions using different temperature levels. Taking plastic viscosity as the observation metric, the temperature sensitivity differences between solid concentrations of 70% and 74% were compared. As shown in Figure 15, at 20 °C, the differences in plastic viscosity across various cement–sand ratios were minimal. However, as the temperature rose above 40 °C, the plastic viscosity of the 74% concentration samples decreased significantly more than that of the 70% group. Furthermore, the inhibitory effect of high temperature on plastic viscosity became more pronounced with higher cement–sand ratios. Sensitivity to material proportion parameters increases in high-temperature environments. Therefore, parameter combinations should be optimized for temperature gradients in practical applications to enhance model stability under varying temperature conditions.

**Figure 16:**
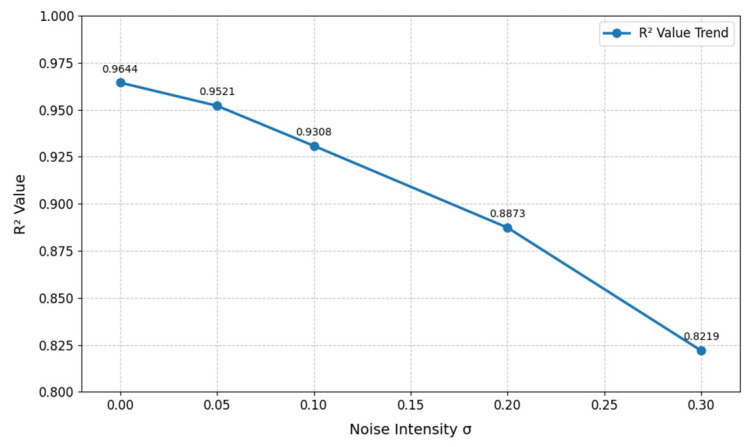
Effect of noise intensity on R^2^ values.

In the sensitivity analysis of sensor noise disturbances, Gaussian noise of varying intensities was added to the pressure data (to simulate measurement errors) to analyze the trend in the R^2^ value of the model output. As shown in Figure 16, when there is no noise, the R^2^ value is 0.9644, indicating good model fitting performance. As the noise intensity increases, the R^2^ value gradually decreases. When σ = 0.30, the R^2^ value drops to 0.8219, indicating that the higher the noise intensity, the more significant the decline in model fitting accuracy, and strong noise can severely impair model performance. From the perspective of data change magnitude, when the noise intensity increases from 0.00 to 0.05, the decrease in the R^2^ value is relatively small. However, when the noise intensity increases from 0.25 to 0.30, the decrease in the R^2^ value becomes more significant, indicating that the model is more sensitive to high-intensity noise. Once the noise intensity exceeds a certain threshold, the degradation of model accuracy accelerates. This phenomenon suggests that during actual data collection, it is essential to strictly control noise intensity at a low level (σ ≤ 0.10) to prevent model performance from deteriorating sharply due to noise accumulation.

## 5. Conclusions

In pipeline transportation systems, conventional methods for calculating resistance loss often employ single-factor analysis, leading to significant deviations between simulation results and actual operational conditions. These approaches struggle to effectively address rheological characteristics under complex multi-factor coupling effects. With the advent of Industry 4.0, artificial intelligence and data-driven technologies have emerged as crucial tools for enhancing system efficiency and accuracy. To address these challenges, this study proposes an innovative approach based on the FlowTransKAN framework. By integrating generative artificial intelligence technology, our approach employs a flow-based model to synthesize physically consistent data, thereby expanding the sample space while preserving fundamental physical principles. The framework incorporates B-spline basis functions from the KAN network to enable sophisticated nonlinear feature extraction. Furthermore, leveraging the Transformer’s multi-head attention mechanism allows the model to effectively capture spatio-temporal correlations within pipeline pressure sequences. This integrated architecture demonstrates significant improvements in the accuracy of resistance loss calculations compared to conventional methods. In the Industry 4.0 paradigm, the integration of smart sensors and IoT technologies has enabled the collection of richer real-time data streams for pipeline transportation systems. When combined with cloud computing infrastructure and advanced big data analytics, these technological advancements significantly enhance model performance in addressing complex, multi-variable nonlinear problems. Our data-driven methodology equips the system with more precise predictive and optimization capabilities, enabling robust adaptation to dynamic and uncertain operating conditions. Experimental results demonstrate that the FlowTransKAN framework outperforms conventional models across multiple performance metrics, particularly excelling in scenarios characterized by limited training data and complex parameter interactions, where it maintains superior prediction accuracy. This shows that FlowTransKAN has a wide range of application prospects in engineering fields such as mine filling and can effectively solve the problem of resistance loss under multi-parameter and multi-variable conditions that traditional methods cannot cope with, providing a new solution for intelligent pipeline transportation system in Industry 4.0 environments.

In practical mining operations, environmental factors, including temperature fluctuations, humidity variations, and safety constraints, frequently compromise raw data quality. These measurement errors are particularly challenging in mining environments, where they often evade timely detection. Furthermore, the prohibitive costs associated with repeated measurements significantly increase the economic burden of data acquisition. Consequently, enhancing model adaptability to complex, unstable operational environments emerges as a critical focus for future research. Future studies can further explore the adaptive ability of the FlowTransKAN model under a variety of complex working conditions and combine smart sensors and IoT technology to obtain more high-quality environmental data in real time. By enhancing the accuracy and coverage of data acquisition, the stability and accuracy of the model can be further improved. At the same time, considering the intelligent manufacturing needs driven by Industry 4.0, we can try to add a lightweight neural network architecture in the future, reduce the computational complexity of the model, and improve the real-time response ability.

## Figures and Tables

**Table 1:** Rheological test orthogonal schedule.

Serial Number	Mass Concentration/%	Temperature/°C
1	70%	20
2	70%	30
3	70%	40
4	70%	50
5	70%	60
6	71%	20
7	71%	30
8	71%	40
9	71%	50
10	71%	60
11	72%	20
12	72%	30
13	72%	40
14	72%	50
15	72%	60
16	73%	20
17	73%	30
18	73%	40
19	73%	50
20	73%	60
21	74%	20
22	74%	30
23	74%	40
24	74%	50
25	74%	60

**Table 2:** Some of the experimental setups for the pipeline transportation test.

Name	Specification	Dimensions (mm)	Included Accessories
Upper Storage Bin	Remodel	Height 2500	1 piece of DN300 flange
Plexiglass Segments	Φ108	Length 6000	2 pieces of DN100 flanges
Reducing	Φ325~Φ273	-	1 piece of DN300 flange and 1 piece of DN225 flange
Carbon Steel Pipes	Φ219	Length 6000	2 pieces of DN200 flanges
Mud Pump	DN100	-	-

**Table 3:** Comparison table of ablation experiments.

	MLP	KAN	TransKAN	FlowTransKAN
R^2^	0.8337	0.8788	0.9017	0.9644
MAE	0.7253	0.5733	0.554	0.4703
RMSE	1.54	1.3149	1.1841	0.7126

**Table 4:** Comparison of experimental results.

	R^2^	MAE	RMSE	Training Time
Edgar Buckingham	/	1.263	3.937	N/A
SVM	0.7952	0.7417	1.7091	124 s
Random Forest	0.8445	0.6317	1.489	81 s
BP Neural Networks	0.9076	0.5134	1.1479	374 s
FlowTransKAN	0.9644	0.4703	0.7126	459 s

**Table 5:** FlowTransKAN model parameters for each part.

Model	Key Parameter	Value
Flow-Based Model	Number of Reversible Layers	8
	Hidden Neurons per Subnetwork	128
	Number of Fully Connected Layers	3
KAN Network	Order of B-Splines	3
	Number of Knots	30
Transformer	Number of Attention Heads	8
	Feed-Forward Network Dimension	128

## Data Availability

Data confidentiality was used in this study. For questions, please contact zhangqinyu@stu.ncst.edu.cn.
